# Supplementary material for: HIVprotI: an integrated web based platform for prediction and design of HIV proteins inhibitors
Source: J Cheminform. 2018 Mar 9;10:12. doi: 10.1186/s13321-018-0266-y (PMC5845081; doi:10.1186/s13321-018-0266-y)
Supplement: Supplementary file 2 — Additional file 2. Source code of HIVProtI web server. [file 13321_2018_266_MOESM2_ESM.zip › HIVprotI_Source-code/hivprotI/weka-3-6-6/doc/weka/gui/SortedTableModel.SortContainer.html]

SortedTableModel.SortContainer


---


|  |  |  |
| --- | --- | --- |
| **Overview** \| **Package** \| **Class** \| **Tree** \| **Deprecated** \| **Index** \| **Help** \| **Weka's home** | |  |
| **PREV CLASS**   **NEXT CLASS** | **FRAMES**    **NO FRAMES**     **All Classes** |  |
| SUMMARY: NESTED \| FIELD \| CONSTR \| METHOD | DETAIL: FIELD \| CONSTR \| METHOD |  |


---


## weka.gui Class SortedTableModel.SortContainer

```
java.lang.Object
  weka.gui.SortedTableModel.SortContainer
```

**All Implemented Interfaces:**: java.lang.Comparable<SortedTableModel.SortContainer>

**Enclosing class:**: SortedTableModel

---

``` public static class SortedTableModel.SortContainer extends java.lang.Object implements java.lang.Comparable<SortedTableModel.SortContainer> ```

Helper class for sorting the columns.

---

**Constructor Summary**

`SortedTableModel.SortContainer(java.lang.Comparable value,
int index)`
  
          Initializes the container.


| **Method Summary** | |
| --- | --- |
| `int` | `compareTo(SortedTableModel.SortContainer o)`             Compares this object with the specified object for order. |
| `boolean` | `equals(java.lang.Object obj)`             Indicates whether some other object is "equal to" this one. |
| `int` | `getIndex()`             Returns the original index of the item. |
| `java.lang.Comparable` | `getValue()`             Returns the value to sort on. |
| `java.lang.String` | `toString()`             Returns a string representation of the sort container. |

**Methods inherited from class java.lang.Object**

`getClass, hashCode, notify, notifyAll, wait, wait, wait`

**Constructor Detail**

### SortedTableModel.SortContainer

```
public SortedTableModel.SortContainer(java.lang.Comparable value,
                                      int index)
```

:   Initializes the container.

    **Parameters:**: `value` - the value to sort on: `index` - the original index


**Method Detail**

### getValue

```
public java.lang.Comparable getValue()
```

:   Returns the value to sort on.

    :   **Returns:**: the value

---


### getIndex

```
public int getIndex()
```

:   Returns the original index of the item.

    :   **Returns:**: the index

---


### compareTo

```
public int compareTo(SortedTableModel.SortContainer o)
```

:   Compares this object with the specified object for order. Returns a
    negative integer, zero, or a positive integer as this object is less
    than, equal to, or greater than the specified object. Null is considered
    smallest. If both values are null, then 0 is returned.

    :   **Specified by:**: `compareTo` in interface `java.lang.Comparable<SortedTableModel.SortContainer>`
    :   **Parameters:**: `o` - the object to be compared. **Returns:**: a negative integer, zero, or a positive integer as this object is less than, equal to, or greater than the specified object. **Throws:**: `java.lang.ClassCastException` - if the specified object's type prevents it from being compared to this object.

---


### equals

```
public boolean equals(java.lang.Object obj)
```

:   Indicates whether some other object is "equal to" this one.

    :   **Overrides:**: `equals` in class `java.lang.Object`
    :   **Parameters:**: `obj` - the reference object with which to compare. **Returns:**: true if this object is the same as the obj argument; false otherwise. **Throws:**: `java.lang.ClassCastException` - if the specified object's type prevents it from being compared to this object.

---


### toString

```
public java.lang.String toString()
```

:   Returns a string representation of the sort container.

    :   **Overrides:**: `toString` in class `java.lang.Object`
    :   **Returns:**: the string representation (value + index)


---


|  |  |  |
| --- | --- | --- |
| **Overview** \| **Package** \| **Class** \| **Tree** \| **Deprecated** \| **Index** \| **Help** \| **Weka's home** | |  |
| **PREV CLASS**   **NEXT CLASS** | **FRAMES**    **NO FRAMES**     **All Classes** |  |
| SUMMARY: NESTED \| FIELD \| CONSTR \| METHOD | DETAIL: FIELD \| CONSTR \| METHOD |  |


---
